# Case report: Familial hypoparathyroidism with elevated parathyroid hormone due to an inactivating *PTH* mutation

**DOI:** 10.3389/fendo.2024.1415639

**Published:** 2024-10-07

**Authors:** Noha Mukhtar, Balgees Alghamdi, Meshael Alswailem, Afaf Alsagheir, Ali S. Alzahrani

**Affiliations:** ^1^ Department of Medicine, King Faisal Specialist Hospital & Research Centre, Riyadh, Saudi Arabia; ^2^ Department of Molecular Oncology, King Faisal Specialist Hospital & Research Centre, Riyadh, Saudi Arabia; ^3^ Department of Paediatrics, King Faisal Specialist Hospital & Research Centre, Riyadh, Saudi Arabia

**Keywords:** hypoparathyroidism, familial hypoparathyroidism, hypocalcemia, parathyroid hormone, PTH, mutation

## Abstract

**Introduction:**

So far, only 11 *PTH* mutations have been described as causes of familial isolated hypoparathyroidism (FIH). In this report, we describe a family with FIH but with significant elevation of functionally inactive PTH due to a *PTH* mutation. We also show a positive therapeutic outcome of recombinant human PTH (teriparatide) therapy in one of the siblings who was not well controlled on large doses of calcitriol and calcium replacement therapy.

**Case description:**

The proband is a 34-year-old woman who has a history of chronic severe hypocalcemia (HypoCa) since birth. She and her three brothers (33-year-old male twins, and a 21-year-old male) were diagnosed with pseudohypoparathyroidism type 1b (PHPT 1b) based on the presence of chronic HypoCa (serum Ca 1.6-1.85 mmol/l) since birth associated with significantly elevated plasma PTH levels in the range of 310-564 pg/dl (normal range 10-65) and absence of signs of Albright hereditary osteodystrophy.

**Molecular studies:**

WES showed no pathogenic, likely pathogenic or variants of unknown significance in any known calcium-associated genetic disorder but a bi-allelic variant in the *PTH* itself ((NM_000315.4:c.128G>A, p.Gly43Glu). This was confirmed by Sanger sequencing in the patient and her affected brothers.

**Management:**

Because the patient’s HypoCa was not controlled on large doses of calcitriol and calcium carbonate, a trial of teriparatide 20 mcg SC daily was started and resulted in normalization of calcium, decline in PTH levels and significant improvement in her general wellbeing.

**Conclusion:**

High PTH in the presence of congenital hypocalcemia is not always due to receptor or post-receptor defect and can be due to a biologically inactive mutated PTH. In such cases, treatment with teriparatide may result in stabilization of biochemical profile and improvement in quality of life.

## Introduction

In human, plasma calcium level is tightly regulated by vitamin D and parathyroid hormone (PTH) (1). Hypocalcemia results from acquired or congenital disorders that affect levels or action of these two hormones (2, 3). Vitamin D disorders include acquired disorders such as vitamin D deficiency and hereditary disorders causing resistance to its action such as vitamin D resistant rickets. Similarly, hypocalcaemia may result from acquired or congenital/hereditary causes of hypoparathyroidism (e.g. familial isolated hypoparathyroidism) or resistance to its actions (pseudohypoparathyrodism, PHPT) (2, 3).

Familial isolated hypoparathyroidism (FIH) is caused by structural defects in parathyroid gland development or genetic defects causing low-level or biologically inactive PTH (4). Structural parathyroid defects are rare and associated most commonly with loss of function mutations in the glial-cell missing-2 gene (*GCM2*) (OMIM 618 883), an important transcription factor for parathyroid gland development (4–6). The causes of low PTH in the absence of parathyroid gland structural defects include activating mutations in the calcium-sensing receptor (*CASR*) leading to suppression of PTH secretion (autosomal dominant hypocalcemia type 1; OMIM 601 198) (7, 8) or in the alpha subunit of the G11 protein (*GNA11*, autosomal dominant hypocalcemia type 2; OMIM 615 361) (9, 10) which mediates the intracellular signaling of PTH action. A rare form of FIH is due to mutations in the *PTH* gene itself (11). These mutations may lead to impaired synthesis of PTH (12), endoplasmic reticulum-mediated stress response leading to apoptosis of parathyroid cells (13–17) or secretion of biologically inactive form of PTH (18–20). So far, only 11 such mutations have been described in the literature (11, 12, 14, 16–24). In this report, we describe a family with four affected siblings who were diagnosed for many years as cases of PHPT type 1b because they had chronic hypocalcemia and elevated levels of PTH. Recently, whole exome sequencing (WES) revealed that these patients are not cases of PHPT but their chronic hypocalcemia is due to a loss-of-function *PTH* mutation leading to hypersecretion of biologically inactive PTH. In one of these siblings who suffered from severe resistant symptomatic hypocalcemia, treatment with recombinant PTH (Teriparatide) resulted in normalization of serum calcium and phosphate with resolution of symptoms.

## Patients and methods

### Patients

#### Sibling 1 (Proband)

**Figure 1:**
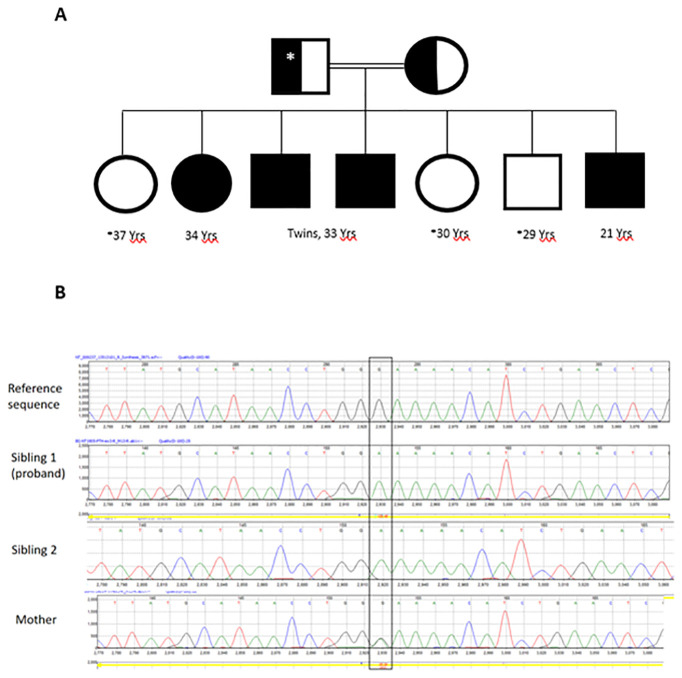
The family pedigree **(A)**. Open circles and squares are normal subjects without symptoms but not tested biochemically or genetically. The asterisk denotes no genetic testing was performed. Father was not tested but presumed to be heterozygous for the PTH variant identified in the siblings. Chromatograms of PTH exon 3 Sanger sequencing **(B)** showing normal sequence (upper panel), a bi-allelic variant (c.128G>A; p.G43E) in the proband and sibling 1 (middle panels) and the same variant in a mon-allelic form in the mother (lower panel).

The proband is a 34-year-old woman, one of a 7-sibling family (**Figure 1A**), who has a history of chronic hypocalcemia since birth with several admissions for severe hypocalcemia and recurrent seizures during childhood. She and her three affected brothers (33-year-old male twins and a 21-year-old male) were diagnosed to have PHPT type 1b based on the presence of chronic hypocalcemia (serum Ca 1.6-1.85 mmol/l) since birth associated with significantly elevated plasma PTH levels in the range of 310-564 pg/dl (normal range 10-65) and absence of signs of Albright hereditary osteodystrophy (AHO). When evaluated at our center at age 34 years, her height was 153 cm, weight 73 Kg, and BMI 31.2 Kg/m^2^. She had no dysmorphic features, cataract, skeletal deformities, or developmental features suggestive of AHO. She was compliant with her medications (calcium carbonate 600-1200 mg PO BID and calcitriol 1.5 mcg PO OD). Laboratory workup (**Table 1**) showed: PTH 569 ng/L (normal range 15-65), calcium 1.90 mmol/l (2.1-2.6), phosphate 1.52mmol/L (0.8-1.4), magnesium 0.63 mmol/L (0.7-1.0), albumin 38.3 g/L (35-45), Alkaline phosphatase 80 U/L (46-122), eGFR >60 ml/min/1.73 m^2^, creatinine 90 umol/L (46-96) and 25-hydroxyvitamin D 55 nmol/l (50-120). Daily excretion of calcium was normal at 3.38 mmol/day (2.5-8.0) and urine phosphate 14.3 mmol/day (11-32). An ultrasound of the kidneys suggested medullary nephrocalcinosis but no renal stones. Skeletal X-rays showed increased bone density only without deformities or fractures. Bone densitometry was super normal with Z score of +3.5 in the lumbar spine and +2.7 in femoral neck. An ultrasound of parathyroid glands revealed non-visible parathyroid glands and no parathyroid adenomas. An MRI of the brain revealed no evidence of brain deformities or calcification.

**Table 1:** Clinical and biochemical data of the affected family members.

Features	Subject 1	Subject 2	Subject 3	Subject 4
Current age (years)	34	33	33	21
Sex	Female	Male	Male	Male
Weight (Kg)	80	75	80.2	51
Height (cm)	153	167	167	161
BMI (Kg/m^2^)	34.2	26.9	29	19.7
Creatinine (46-96) umol/l	90	165	150	126
Ca (2.1-2.6) mmol/l	1.90	1.79	1.78	2.03
PO4 (0.8-1.46) mmol/l	1.52	1.79	2.02	1.29
Albumin (35-45) g/l	38.7	47	48.3	47.5
Mg (0.7-1.0) mmol/l	0.63	0.74	0.82	0.81
Alkaline phosphatase U/l	80	23	27	72
PTH (15-65) ng/l	569	504	454	803
25-OH Vitamin D (50-120) nmol/l	55	52	47	36

#### Sibling 2

A 33-year-old male was also diagnosed during childhood with PHPT type 1b when he presented with symptomatic hypocalcemia and seizure soon after birth. He was treated with different doses of calcium carbonate (1200 mg/day and calcitriol 2-3 mcg/day at time of evaluation) with inadequate control of his symptoms and frequent recurrent hypocalcemia. At age 29 years, he presented with increasing stiffness of the lower trunk and thigh muscles and an inability to walk. His evaluation revealed a new diagnosis of stiff person syndrome based on neuromuscular evaluation and extremely high anti-glutamic acid decarboxylase 65 (GAD65) antibodies (> 2000 units/l). He was treated with diazepam 10 mg Q6hrs and baclofen 20 mg q6hr. His clinical status continued to worsen and incapacitated his daily life. He received intravenous immunoglobulin (IVIG) therapy and eventually underwent autologous hematopoietic stem cell transplantation in August 2022. He improved and became ambulatory again but with recurrent tetany-like episodes and chronic muscle spasticity. He had evidence of chronic kidney disease (CKD) stage 3. Full workup was negative for proteinuria and hematuria. An ultrasound of the kidneys revealed normal size kidneys without stones, nephrocalcinosis or obstruction. A kidney biopsy failed to indicate a clear cause of his CKD with no evidence of glomerular abnormality but mild interstitial fibrosis and tubular atrophy. Apart from moderate muscle spasticity, his clinical examination was unremarkable and did not reveal any dysmorphic or skeletal features or signs of AHO. Laboratory evaluation is summarized in **Table 1**. MRI of the brain showed normal brain structures including basal ganglia with no evidence of calcifications. He has been treated with calcium carbonate 1200 mg TID and calcitriol 1-1.5 mcg daily with fluctuating calcium levels ranging between 1.81-2.10 mmol/l with recurrent symptoms of muscle spasms. He has not yet been treated with Teriparatide.

#### Sibling 3

RA is a 33-year-old man, a twin brother of subject 2. He presented similarly with neonatal seizure and hypocalcemia and has been on calcium carbonate 1200 mg TID and calcitriol 1.5-2 mcg daily with inadequate control of his hypocalcemia and its symptoms. His biochemical parameters are summarized in **Table 1**. Similar to his twin brother, he developed CKD stage 3 of unclear cause. An ultrasound of the kidneys showed normal-sized kidneys. A small cyst of 2 cm in size and a focus of calcification was seen in the left kidney. Renal biopsy revealed only minimal interstitial fibrosis and tubular atrophy. Unlike his twin brother, he did not develop features of stiff person syndrome. He also was not treated with teriparatide.

#### Sibling 4

A 21-year-old man with a history of symptomatic hypocalcemia since birth presenting with muscle spasms and seizures. His physical examination was unremarkable. He has been treated with calcitriol 1-1.5 mcg daily and calcium carbonate 600-1200 mg TID. His biochemical values are summarized in **Table 1**. Like his two brothers (subjects 2 and 3), he has impaired renal function. He has not yet been tested for PTH or other mutations.

### Biochemical evaluation

We used a double antibody sandwich electrochemiluminescence immunoassay (Elecsys PTH, Roche Diagnostics GmbH, Mannheim, Germany, Catalogue No. 07251068190) on Cobas e 402 machine. The antibodies used in this assay are reactive with epitopes in the amino acid regions 26-32 and 37-42. These two epitopes are downstream of the mutation found in this family, which is located at the amino acid 12 of the intact 1-84 PTH polypeptide (amino acid 43 of the preproPTH). Calcium, Phosphate, albumin, alkaline phosphatase, and 25-OH Vitamin D were measured using the multichannel analyser Cobas e 402 (Roche Diagnostics GmbH, Mannheim, Germany).

### Molecular evaluation

An Institutional Review Board and ethics committee approval was obtained from the Office of Research Affairs of King Faisal Specialist Hospital and Research Centre, Riyadh, Saudi Arabia (RAC# 2130012). Informed consents were signed by the patients. WES was done on DNA isolated from peripheral leucocytes of siblings 2 and 3 and WES was performed as previously described (25). Briefly, we used an Illumina NovaSeq 6000 platform as follows: DNA pair-end fragment library was prepared according to Illumina WES protocol using the TruSeq^®^ Exome Kit (Catalog number 20020615, Illumina Inc. San Diego, CA, USA) with a target insert size of 150 bp. DNA sequencing was performed using the Illumina NovaSeq 6000 platform (S2 reagent kit, 200 cycles configuration, and target sequencing depth of 50X. The identified variant was confirmed by Sanger sequencing of DNA isolated from peripheral leucocyte of the proband and subject 2 and their mother using primers and PCR conditions as previously described (17).

## Results

### Molecular studies

WES showed no pathogenic, likely pathogenic or variants of unknown significance in *GNAS, PTHR, CASR, VDR, CYP27B1, AIRE, AP2S1, CHD7, CYP24A1, FAM111A, GATA3, GCM2, GNA11, GNAS, HADHA, HADHB, PDE4D, PRKAR1A, PTH1R, SOX3, STX16, TBCE, and TBX1.* However, a variant in the *PTH* itself (NM_000315.4: c.128G>A, p.Gly43Glu) was found. This was confirmed by Sanger sequencing in the proband and sibling 2 (**Figure 1B**). Sibling 3 also had the same variant on WES. The *PTH* variant was also present in their mother in a mono-allelic form (**Figure 1B**). This variant was reported before and assessed to be likely pathogenic or likely damaging by HGMD and several in silico analysis tools including Polyphen2, MutPred, PROVEAN, DEOGEN2, MVP and SIFT4G.

As a secondary finding, the two twin brothers (Subject 2 and 3) who had WES showed a heterozygous variant in *COQ8B* (NM_024876.3, c.532C>T, p.Arg178Trp). In a homozygous form, this variant was reported in several studies to cause steroid-resistant nephrotic syndrome.

### Clinical assessment and management

**Figure 2:**
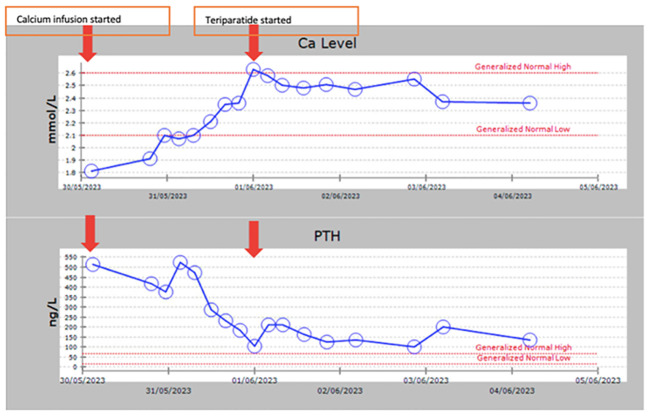
A graph showing calcium and PTH levels following continuous intravenous calcium infusion and overtime during subcutaneous chronic daily teriparatide therapy.

Due to the extremely high PTH level with hypocalcemia, and in order to assess the clinical and biochemical response to calcium replacement, we admitted the proband (Sibling 1) and started her on a continuous intravenous calcium infusion titrated according to serum calcium levels monitored every 4 hours (**Figure 2**). An anticipated decline in PTH level was observed (from 514 to 185 ng/L) as calcium levels increased and normalized (2.35 mmol/l). Calcium infusion was discontinued and she was treated with recombinant human PTH hormone (teriparatide 20 mcg SC OD). Calcitriol dose was decreased to 1 mcg daily and calcium carbonate to 600 mg BID. On this therapy, she maintained a normal calcium level and her PTH level further decreased to 99 ng/L after only 2 doses of teriparatide initiation (**Figure 2**). She has been on this treatment for about 9 months. Her calcium level remained normal during this period and she has no symptoms of hypocalcemia. Her quality of life has significantly improved and she did not need hospital admissions since she was started on teriparatide. Creatinine remained stable without changes on teriparatide. Other siblings have not yet been treated with teriparatide.

## Discussion

In this report, we describe a family with a very rare mechanism of familial hypocalcemia due to a *PTH* mutation. This mutation led to non-functional PTH causing hypocalcemia and triggering a secondary rise of biologically inactive PTH. In the era before next-generation sequencing, these cases would be most often diagnosed as PHPT type Ib and this was the case in our family. It is possible that many similar cases have been labeled as such and this may have even contributed to a delay in understanding the molecular basis of PHPT 1b. Another interesting aspect of our family is the excellent therapeutic response to the recombinant human PTH (Teriparatide) in the proband, which is consistent with a ligand (PTH) defect rather than a PTH receptor or post-receptor mechanism in which case, the response is unlikely to be as robust if any. This also suggests that Teriparatide might be a good option for these cases, especially those who cannot be controlled on active vitamin D and calcium supplement therapy.

PTH is synthesized in the parathyroid cells as preproPTH and its gene (OMIM 168 450) was mapped to chromosome 11.p15.3 (26). The pre-sequence is a hydrophobic 25-amino acid sequence followed by a 6 positively-charged pro-sequence (27). The pre-sequence is cleaved off in the rough endoplasmic reticulum while pro-sequence is processed and removed in the Golgi apparatus (28). The mature molecule is an 84-amino acid polypeptide secreted by the parathyroid cells, circulates in the blood and regulates the plasma calcium level and renal phosphate excretion (29). *PTH* mutations are very rare (11). Only 11 such mutations have been described in the literature (**Table 2**) (13–15, 17–22, 24). Unlike our case, most of the previously described cases had low PTH levels in the presence of hypocalcemia. Arnold A et al. described a mono-allelic mutation at codon 18 of the preproPTH in a 20-year-old man who presented with hypocalcemia and low PTH since the neonatal period (13). This mutation interferes with the processing of the preproPTH in the endoplasmic reticulum. The family study showed a brother with a similar condition and an inadequate PTH response to hypocalcemia in the father only suggesting an autosomal dominant inheritance (13). A subsequent study described a 24-year old girl who also presented with neonatal hypocalcemia and seizure and found to have undetectable PTH due to a mutation at codon 23 of the preproPTH leading to inefficient processing of the prehormone (23). This position is -3 residue in the signal peptide and the authors speculated that this may interfere with signal peptide cleavage leading to degradation of preproPTH in the endoplasmic reticulum (23). Unlike the previous case, family studies showed the parents to be heterozygous for the same mutation suggesting an autosomal recessive inheritance (23).

**Table 2:** Summary of previously reported patients with familial isolated hypoaparathyroidsm due to PTH mutations.

Reference	Presentation	Age/sex	Family History/Inheritance^	Ca	PO4	PTH**	Mutation	
				mmol/l	mmol/l	Value (range)	*Nucleotide*	*Amino acid*
Arnold A. et al. (13)	Neonatal hypoCa, seizure	20Y/F	A brother (AD) Father had an inadequate PTH response to hypocalcemia	1.8	1.5	0.46 ng/ml (0.5-1.6)	c.52T>C	p.Cys18Arg
Sunthornthepvarakul T. et al. (23)	hypoCa, seizure	20Y/F	2 sisters and a niece (AR)	1.5	2.9	Undetectable	c.67T>C	p.Ser23Pro
Ertl D. et al. (22)	Tetany	4M/F	2 unaffected siblings (one of them and the parents were heterozygous for the mutation (AR)	1.46	3.71	2.1 pg/ml (15-65)	c.68C>A	p.Ser23Term
Tomar N. et al. (24)	hypoCa, seizure	18Y/F	No family history of FIH, a heterozygous mutation (AD)	1.82	2.23	5.29 pg/ml (15-65)	c.2T>C (hetero)	p.Met1_Asp6del
Lee S. et al. (18)	hypoCa, seizure	55Y/M 50Y/F 47Y/F	3 affected siblings (AR)	1.2 1.77 1.62	2.62 1.55 2.0	< 5 pg/ml* (10-65) **1455** pg/ml (9-39) **679** pg/ml (9-39)	c.166C>T	P.Arg56Cys
Cinque L. et al. (14)	Flexor hypertonia	6D/M	3 members of 3 generations are affected, the mutation is heterozygous (AD)	1.67	3.33	4.8 pg/ml (15-65)	c.41T>A (Hetero)	p.Met14Lys
Dixon J. et al. (21)	Symptomatic hypoCa	4Y/F	Parents and sisters presumed to be heterozygous for the variant with symptoms (? AR, dose effect)	1.12	2.71	<0.2 pmol/l (1.7-7.3)	c.68C>A	p.Ser23Term
Gild M. et al. (19)	hypoCa	54Y/F 47Y/F	2 sisters affected (AR)	2.25 2.35	1.50 1.40	**185** pmol/l (1.6-6.9) **150** pmol/l (1.6-6.9)	c.94T>C	p.Ser32Pro
Hanna P. et al. (17)	hypoCa, seizure	2M/F	None affected but parents are consanguineous and heterozygous for the same PTH variant (AR)	1.17	1.65	**110** pg/ml at 2 months of age, **472** pg/ml at 9 years and **359** pg/ml at 14.6 years	c.94T>C	p.Ser32Pro
Hawkes C. et al. (20)	hypoCa, seizure	8d/M 11Y/M	No family Hx of FIH, parents tested negative for the variant, the variant was heterozygous (AD) 13 members of 3 generations are affected, a homozygous mutation (AR)	1.3 1.7	4.1 2.61	0.6 pmol/l (1.1-6.9) **23.8** pmol/l (1.6-6.9)	c.46_47delinsAA c.128G>A	p.Ala16Lys p.Gly43Glu

^AD, autosomal dominant; AR, autosomal recessive.

*Measured by an assay that detects only the amino-terminal segment. PTH in the other two siblings used an assay that measures the full-length PTH (1-84).

**Most cases of FIH had low PTH levels, and some cases had elevated PTH levels (in bold).

Ertl D. et al., Tomar N. et al., Cinque L. et al. and Dixon J. et al. described similar cases (**Table 2**) who presented mostly in the neonatal or early childhood periods with symptomatic hypocalcemia and low to undetectable PTH levels (14, 21, 22, 24). They were found to have missense or nonsense mutations in exon 1 or exon 2 of the preproPTH gene (14, 21, 22, 24). Except for one heterozygous mutation (c.2T>C) reported by Tomar N. et al. (24), all other mutations were homozygous suggesting an autosomal recessive inheritance. The mechanisms by which these mutations interfere with PTH action are presumed to be either by interference with preproPTH processing and its degradation in the endoplasmic reticulum or by loss of function of the 1-84 amino acid PTH due to amino acid change in the mature PTH (1-84) (20).

In contrast to the above-mentioned reports in which patients presented with a classic biochemical profile of hypoparathyroidism with hypocalcemia and low to undetectable PTH levels, a few reports described patients with elevated plasma levels of biologically inactive PTH molecule (17, 19, 20). Gild et al., described two sisters who presented with neonatal hypocalcemia (19). Their PTH levels were low normal until mid-adulthood when they started to increase progressively reaching > 100-fold (19). They had a *PTH* mutation (c.94T>C, p.Ser32Pro). The authors speculated that the increasing PTH levels were due to parathyroid hyperplasia that gradually developed over time due to chronic stimulation of the parathyroid glands by hypocalcemia (19). In a follow up study, Hanna P. et al. described the mechanism by which this mutation in the first codon of the mature PTH (c.94T>C, p.S32P) caused hypocalcemia with elevated levels of PTH (17). Three patients of two unrelated families (Two patients from the family described by Glid M et al. (19) described above and one patient from another unrelated family) were found to have a homozygous Serine to Proline mutation affecting the first codon of the mature PTH (1-84). In contrast to the wild type proPTH, the mutant ProPTH was found resistant to cleavage by furin suggesting that this variant impairs preproPTH processing (17). This was further supported by finding high levels of the proPTH (-6-84) in the affected patients (17). Hawkes et al. described an Arab family with several members presenting with neonatal hypocalcemia but with elevated levels of PTH. Genetic testing revealed a *PTH* exon 3 (c.128G>A; p.G43E) mutation producing a biologically inactive PTH which was elevated due to secondary stimulation by hypocalcemia (20).

Our patients have the same mutation of the family that was described by Hawkes et al. (20). Both families are of Arab descent but to our knowledge, they are unrelated. The first family is from the United Arab Emirates and our family is from the south part of Saudi Arabia. However, we cannot exclude a remote ancestral common descent. This mutation was extensively characterized in the previous report (20). The response to calcium infusion and teriparatide in our patient and the previously described family shows normal physiological responsiveness of parathyroid cells to the increasing plasma calcium level and normal PTH receptor and post-receptor response mechanisms. To our knowledge, our patient is the first patient with a *PTH* mutation causing hypoparathyroidism to be treated for a long time with Teriparatide with remarkable subjective and biochemical improvement of her condition over many months and after several years of uncontrolled symptomatic hypocalcemia. This suggests that this therapy is physiologically and therapeutically appropriate for such cases, especially those who are not well controlled on active vitamin D and calcium supplements.

Our family has a number of additional interesting aspects that is worth briefly discussing. The affected family members include monozygotic twin brothers. This is the first report of twins with FIH. Both are affected with FIH but one of them also developed stiff person syndrome suggesting that it is a coincidental rather than related condition to FIH although both conditions cause muscle spasms. Stiff man syndrome is an extremely rare syndrome characterized by stiffness and pain mostly of the trunk muscles and lower limbs (30). It is believed to be an autoimmune disease characterized by antibodies against the inhibitory GABAergic pathways (30, 31). Anti GAD antibodies are the most commonly identified antibodies (32). Immunomedullary therapies including glucocorticoids, IVIG, plasmapheresis and rituximab have been used in its therapy (30). Autologous hematopoietic stem cell transplantation was introduced in 2014 as treatment of two cases (33) and has been increasingly used since then with promising results for cases of refractory stiff person syndrome (34, 35).

Another interesting aspect of our family is the development of CKD in three affected brothers. Although nephrocalcinosis has been described in FIH but renal impairment has not been described. The cause of CKD is these siblings is unclear but the twin brothers are heterozygous for a mutation in a recently described nephrotic syndrome-associated gene, COQ8B (NM_024876.3, c.532C>T, p.Arg178Trp). The other two siblings (Subject 1 and 4) did not have WES and were not tested for this mutation. Whether this mutation has contributed to the CKD in these patients or not is unclear. *COQ8B* along with many other genes (COQ2, COQ4, COQ6, PDSS1, PDSS2, and ADCK3) are involved in the biosynthetic pathway of the coenzyme Q10 (CoQ10). This coenzyme, also called ubiquinone, is a lipid-rich compound present essentially in all cell membranes and plays an important antioxidant role in addition to its contribution to the electron transport from complex I and II to complex III in the respiratory chain cycle. Previous studies have shown that homozygous mutations in this gene and other CoQ10 genes cause steroid-resistant nephrotic syndrome (36–38). The mutation found in our patients have been reported and well characterized as pathogenic in many studies (36–38). However, in all previous studies with nephrotic syndrome, the mutation was bi-allelic. In our patients, it is mono-allelic and whether it contributed to renal impairment as a result of haploinsufficiency or not remains unclear. In addition, our patients did not have any proteinuria and renal biopsy from one of them showed no glomerular damage but only some interstitial fibrosis and tubular atrophy.

In summary, we have described a new family with FIH who presented with symptomatic hypocalcemia since birth. Unexpectedly, their plasma PTH levels were very high. This led to their misdiagnosis of PHPT type 1b for many years before WES disclosed the underlying PTH mutation, which caused hypocalcemia and secondary hyperparathyroidism of biologically inactive PTH. In all affected patients, the condition remained inadequately controlled with large doses of active vitamin D and calcium supplements but was well responsive to Teriparatide in one of the affected siblings. This suggests that PTH analogues such as teriparatide might be considered in the treatment of patients with FIH due to PTH mutations, especially in severe resistant cases. Other interesting aspects of this family are the occurrence of FIH in twin brothers, the development of stiff man syndrome in one of the twin brothers and the occurrence of CKD in three affected brothers, potentially contributed to by another heterozygous mutation in an unrelated gene (COQ8B).

## Data Availability

The primary data related to the work presented is included in this article. Additional data are available upon request from the corresponding authors.

## References

[1] Mallette LE . Regulation of blood calcium in humans. Endocrinol Metab Clinics North America. (1989) 18:601–10. doi: 10.1016/S0889-8529(18)30355-4 2673764

[2] Cooper MS Gittoes NJ . Diagnosis and management of hypocalcaemia. Bmj. (2008) 336:1298–302. doi: 10.1136/bmj.39582.589433.BE PMC2413335 18535072

[3] Shaw N . A practical approach to hypocalcaemia in children. Endocr Dev. (2009) 16:73–92. doi: 10.1159/000223690 19494662

[4] Newey PJ Hannan FM Wilson A Thakker RV . Genetics of monogenic disorders of calcium and bone metabolism. Clin Endocrinol (Oxf). (2022) 97:483–501. doi: 10.1111/cen.14644 34935164 PMC7614875

[5] Ding C Buckingham B Levine MA . Familial isolated hypoparathyroidism caused by a mutation in the gene for the transcription factor gcmb. J Clin Invest. (2001) 108:1215–20. doi: 10.1172/jci13180 PMC209530 11602629

[6] Yi HS Eom YS Park Ie B Lee S Hong S Jüppner H . Identification and characterization of C106r, a novel mutation in the DNA-binding domain of gcmb, in a family with autosomal-dominant hypoparathyroidism. Clin Endocrinol (Oxf). (2012) 76:625–33. doi: 10.1111/j.1365-2265.2011.04256.x PMC3701386 22066718

[7] Thakker RV . Diseases associated with the extracellular calcium-sensing receptor. Cell Calcium. (2004) 35:275–82. doi: 10.1016/j.ceca.2003.10.010 15200151

[8] Hannan FM Kallay E Chang W Brandi ML Thakker RV . The calcium-sensing receptor in physiology and in calcitropic and noncalcitropic diseases. Nat Rev Endocrinol. (2018) 15:33–51. doi: 10.1038/s41574-018-0115-0 30443043 PMC6535143

[9] Nesbit MA Hannan FM Howles SA Babinsky VN Head RA Cranston T . Mutations affecting G-protein subunit α11 in hypercalcemia and hypocalcemia. N Engl J Med. (2013) 368:2476–86. doi: 10.1056/NEJMoa1300253 PMC3773604 23802516

[10] Mannstadt M Harris M Bravenboer B Chitturi S Dreijerink KM Lambright DG . Germline mutations affecting Gα11 in hypoparathyroidism. N Engl J Med. (2013) 368:2532–4. doi: 10.1056/NEJMc1300278 PMC3750735 23802536

[11] Lee JH Davaatseren M Lee S . Rare pth gene mutations causing parathyroid disorders: A review. Endocrinol Metab (Seoul). (2020) 35:64–70. doi: 10.3803/EnM.2020.35.1.64 32207265 PMC7090289

[12] Parkinson DB Thakker RV . A donor splice site mutation in the parathyroid hormone gene is associated with autosomal recessive hypoparathyroidism. Nat Genet. (1992) 1:149–52. doi: 10.1038/ng0592-149 1302009

[13] Arnold A Horst SA Gardella TJ Baba H Levine MA Kronenberg HM . Mutation of the signal peptide-encoding region of the preproparathyroid hormone gene in familial isolated hypoparathyroidism. J Clin Invest. (1990) 86:1084–7. doi: 10.1172/JCI114811 PMC296835 2212001

[14] Cinque L Sparaneo A Penta L Mencarelli A Rogaia D Esposito S . Autosomal dominant pth gene signal sequence mutation in a family with familial isolated hypoparathyroidism. J Clin Endocrinol Metab. (2017) 102:3961–9. doi: 10.1210/jc.2017-00250 28938448

[15] Datta R Waheed A Shah GN Sly WS . Signal sequence mutation in autosomal dominant form of hypoparathyroidism induces apoptosis that is corrected by a chemical chaperone. Proc Natl Acad Sci U.S.A. (2007) 104:19989–94. doi: 10.1073/pnas.0708725104 PMC2148410 18056632

[16] Karaplis AC Lim SK Baba H Arnold A Kronenberg HM . Inefficient membrane targeting, translocation, and proteolytic processing by signal peptidase of a mutant preproparathyroid hormone protein. J Biol Chem. (1995) 270:1629–35. doi: 10.1074/jbc.270.4.1629 7829495

[17] Hanna P Khatri A Choi S Brabant S Gild ML Piketty ML . Homozygous ser-1 to pro-1 mutation in parathyroid hormone identified in hypocalcemic patients results in secretion of a biologically inactive pro-hormone. Proc Natl Acad Sci U.S.A. (2023) 120:e2208047120. doi: 10.1073/pnas.2208047120 36795755 PMC9974466

[18] Lee S Mannstadt M Guo J Kim SM Yi HS Khatri A . A homozygous [Cys25]Pth(1-84) mutation that impairs pth/pthrp receptor activation defines a novel form of hypoparathyroidism. J Bone Miner Res. (2015) 30:1803–13. doi: 10.1002/jbmr.2532 PMC4580526 25891861

[19] Gild ML Bullock M Luxford C Field M Clifton-Bligh RJ . Congenital hypoparathyroidism associated with elevated circulating nonfunctional parathyroid hormone due to novel pth mutation. J Clin Endocrinol Metab. (2020) 105:2408–12. doi: 10.1210/clinem/dgaa279 32421798

[20] Hawkes CP Al Jubeh JM Li D Tucker SE Rajiyah T Levine MA . Novel pth gene mutations causing isolated hypoparathyroidism. J Clin Endocrinol Metab. (2022) 107:e2449–e58. doi: 10.1210/clinem/dgac086 PMC9113798 35165722

[21] Dixon J Miller S . Successful pregnancies and reduced treatment requirement while breast feeding in a patient with congenital hypoparathyroidism due to homozygous C.68c>a null parathyroid hormone gene mutation. BMJ Case Rep. (2018) 2018:bcr-2017-223811. doi: 10.1136/bcr-2017-223811 PMC5976074 29804071

[22] Ertl DA Stary S Streubel B Raimann A Haeusler G . A novel homozygous mutation in the parathyroid hormone gene (Pth) in a girl with isolated hypoparathyroidism. Bone. (2012) 51:629–32. doi: 10.1016/j.bone.2012.06.009 22722080

[23] Sunthornthepvarakul T Churesigaew S Ngowngarmratana S . A novel mutation of the signal peptide of the preproparathyroid hormone gene associated with autosomal recessive familial isolated hypoparathyroidism*. J Clin Endocrinol Metab. (1999) 84:3792–6. doi: 10.1210/jcem.84.10.6070 10523031

[24] Tomar N Gupta N Goswami R . Calcium-sensing receptor autoantibodies and idiopathic hypoparathyroidism. J Clin Endocrinol Metab. (2013) 98:3884–91. doi: 10.1210/jc.2013-2158 23873991

[25] Alghamdi B Al-Hindi H Murugan AK Alzahrani AS . Thyroid cancer, neuroendocrine tumor, adrenal adenoma, and other tumors in a patient with a germline pms1 mutation. J Endocr Soc. (2023) 7:bvad035. doi: 10.1210/jendso/bvad035 37143695 PMC10153420

[26] Tonoki H Narahara K Matsumoto T Niikawa N . Regional mapping of the parathyroid hormone gene (Pth) by cytogenetic and molecular studies. Cytogenet Cell Genet. (1991) 56:103–4. doi: 10.1159/000133059 1672845

[27] Habener JF Rosenblatt M Kemper B Kronenberg HM Rich A Potts JT Jr . Pre-proparathyroid hormone; amino acid sequence, chemical synthesis, and some biological studies of the precursor region. Proc Natl Acad Sci U.S.A. (1978) 75:2616–20. doi: 10.1073/pnas.75.6.2616 PMC392613 96437

[28] Habener JF Amherdt M Ravazzola M Orci L . Parathyroid hormone biosynthesis. Correlation of conversion of biosynthetic precursors with intracellular protein migration as determined by electron microscope autoradiography. J Cell Biol. (1979) 80:715–31. doi: 10.1083/jcb.80.3.715 PMC2110354 457765

[29] Gardella T Jüppner H Brown E Kronenberg H Potts J Jr . Parathyroid hormone and parathyroid hormone receptor type 1 in the regulation of calcium and phosphate homeostasis and bone metabolism. Endocrinology. (2016) 1:969–90. doi: 10.1016/b978-0-323-18907-1.00056-1

[30] Vlad B Wang Y Newsome SD Balint B . Stiff person spectrum disorders-an update and outlook on clinical, pathophysiological and treatment perspectives. Biomedicines. (2023) 11:2500. doi: 10.3390/biomedicines11092500 37760941 PMC10525659

[31] Gorin F Baldwin B Tait R Pathak R Seyal M Mugnaini E . Stiff-man syndrome: A gabaergic autoimmune disorder with autoantigenic heterogeneity. Ann Neurol. (1990) 28:711–4. doi: 10.1002/ana.410280518 2260859

[32] Solimena M Folli F Denis-Donini S Comi G Pozza G De Camilli P . Autoantibodies to glutamic acid decarboxylase in a patient with stiff-man syndrome, epilepsy, and type I diabetes mellitus. New Engl J Med. (1988) 318:1012–20. doi: 10.1056/NEJM198804213181602 3281011

[33] Sanders S Bredeson C Pringle CE Martin L Allan D Bence-Bruckler I . Autologous stem cell transplantation for stiff person syndrome: two cases from the ottawa blood and marrow transplant program. JAMA Neurol. (2014) 71:1296–9. doi: 10.1001/jamaneurol.2014.1297 25155372

[34] Georges GE Bowen JD Pearlman M Wundes A von Geldern G Kraft GH . Autologous hematopoietic stem cell transplantation may be highly effective treatment for severe stiff person syndrome. Biol Blood Marrow Transplant. (2018) 24:S120. doi: 10.1016/j.bbmt.2017.12.060

[35] Georges G McSweeney P Bowen J Pearlman M Wundes A von Geldern G . Autologous hematopoietic stem cell transplantation may be highly effective treatment for severe, treatment refractory stiff person syndrome (S31. 007). Neurology. (2018) 90:S31.007. doi: 10.1016/j.bbmt.2017.12.060

[36] Ashraf S Gee HY Woerner S Xie LX Vega-Warner V Lovric S . Adck4 mutations promote steroid-resistant nephrotic syndrome through coq10 biosynthesis disruption. J Clin Invest. (2013) 123:5179–89. doi: 10.1172/jci69000 PMC3859425 24270420

[37] Song X Fang X Tang X Cao Q Zhai Y Chen J . Coq8b nephropathy: early detection and optimal treatment. Mol Genet Genomic Med. (2020) 8:e1360. doi: 10.1002/mgg3.1360 32543055 PMC7434746

[38] Alfares A Alfadhel M Wani T Alsahli S Alluhaydan I Al Mutairi F . A multicenter clinical exome study in unselected cohorts from a consanguineous population of Saudi Arabia demonstrated a high diagnostic yield. Mol Genet Metab. (2017) 121:91–5. doi: 10.1016/j.ymgme.2017.04.002 28454995

